# The Multidimensional and Hierarchical Nature of the Questionnaire for Eudaimonic Wellbeing: A Bifactor-ESEM Representation in a Spanish Sample

**DOI:** 10.3389/fpsyg.2020.00422

**Published:** 2020-03-11

**Authors:** Daniela Fadda, M. Paz Quevedo-Aguado, María H. Benavente Cuesta, L. Francesca Scalas

**Affiliations:** ^1^Department of Education, Psychology, Philosophy, University of Cagliari, Cagliari, Italy; ^2^Facultad de Psicología, Universidad Pontificia de Salamanca, Salamanca, Spain

**Keywords:** wellbeing, eudaimonia, ESEM, bifactor analysis, gender invariance

## Abstract

Aim of the present study is to support the multidimensional and hierarchical nature of the Spanish version of Questionnaire for Eudaimonic Wellbeing (QEWB) and to analyze its psychometric properties through the exploratory structural equation modeling (ESEM) framework. Results of the analyses carried out in a sample of university students (*N* = 589, 161 males and 428 females), supported the hypothesized bifactor-ESEM solution, composed by a global eudaimonic wellbeing factor and three specific factors (Sense of Purpose, Purposeful Personal Expressiveness and Effortful Engagement). Specifically, the global factor is relatively well defined by most of the 21 items; moreover, two of the specific factors (Purposeful Personal Expressiveness, Effortful Engagement) keep their own meaningful specificity apart from that explained by the global factor, suggesting that they add information to the eudaimonic wellbeing construct. Regarding criterion-related validity of the QEWB, the global factor was positively correlated with self-esteem. Finally, the scale showed adequate levels of composite reliability and measurement invariance over gender. Differences in latent means showed that girls report higher positive Purposeful Personal Expressiveness and Effortful Engagement than boys, whereas no significant differences were found in relation to global eudaimonic wellbeing. Theoretical implications about the nature of eudaimonic wellbeing are considered.

## Introduction

In subjective wellbeing literature two perspectives have been developed: the edonic perspective, which focuses on the subjective experiences of pleasure (Diener, 1984; Kahneman et al., 1999), and the eudaimonic one, which refers to factors that support fulfillment of human potential and personal growth (Waterman, 1993; Deci and Ryan, 2008).

On one hand, the edonic perspective considers subjective wellbeing as a multidimensional construct characterized by affective factors (e.g., pleasant or unpleasant affective experiences, Bradburn, 1969) and cognitive components including domains such as satisfaction and a global subjective evaluation of life (e.g., Diener et al., 1999). On the other hand, eudaimonia is a more complex concept and it has been operationalized in terms of purpose, autonomy, competence, meaningfulness, social connectedness, self-realization and self-acceptance (e.g., Ryan and Deci, 2001; Baumeister and Vohs, 2002; Huta and Ryan, 2010).

Although both edonia and eudaimonia are positive subjective states, they are distinct constructs (e.g., Waterman, 1993; Delle Fave et al., 2011), and in order to measure wellbeing as a factor associated to contemporary philosophical perspectives of eudaimonia, Waterman et al. (2010) developed the Questionnaire for Eudaimonic Wellbeing (QEWB). The six inter-related components considered by Waterman et al. (2010) to define the items of the questionnaire (“*self-discovery, perceived development of one’s best potentials, a sense of purpose and meaning in life, intense involvement in activities, investment of significant effort, and enjoyment of activities as personally expressive*,” p. 44) included both the aspects of eudaimonic functioning (e.g., self-realization and the pursuit of excellence) and the personal experiences of eudaimonia (e.g., feelings of personal expressiveness).

Consistently with eudaimonic wellbeing literature (e.g., Ryff, 1989; Ryan and Deci, 2001), Waterman et al. (2010) included various aspects of eudaimonic functioning; however, they conducted analyses to confirm a unidimensional structure using a questionable parceling strategy (Marsh et al., 2013), without specifying the theoretical dimensions to which the items belong (see Waterman et al., 2010 for additional details on the scale). Schutte et al. (2013), in a multicultural South African students’ sample, using exploratory factor analysis at the item level supported instead, a multidimensional 3- factor structure characterized by the factors Sense of Purpose, Purposeful Personal Expressiveness, Effortful Engagement, and a 4-factor structure characterized by the factors Sense of Purpose, Effortful Engagement, Engagement in Rewarding Activities, Living from Beliefs; both 3- and 4-factor structures might reflect presumably Waterman et al. (2010)’ criteria used to develop the instrument. However, Schutte et al. found multiple large cross-loadings that could suggest the presence of a possible single overarching dimension.

To clarify the factor structure of the QEWB, Fadda et al. (2017), in an Italian sample of high school students, contrasted the *a priori* one-factor solution suggested by Waterman et al. (2010) and tested using a parceling approach, with the 3- and 4-factor solutions found with exploratory factor analyses by Schutte et al. (2013). The authors also proposed the examination of a bifactor solution through the comparison between confirmatory factor analysis (CFA) and exploratory structural equation modeling (ESEM) frameworks. Results supported a bifactor-ESEM model characterized by a global component of eudaimonic wellbeing (G-factor), but also some specific aspects were found (S-factors: Sense of Purpose, Purposeful Personal Expressiveness, Effortful Engagement).

The ESEM represents an integration of CFA, SEM, and EFA, and it provides a way to use the advantages of exploratory factor analysis for confirmatory and predictive purposes, solving some limitations of CFA models in multidimensional scales that produce biased parameter estimates. Indeed, in the CFA, items are allowed to load only on their pertinent factors, not taking into account multiple cross-loadings present when researchers are interested to assess multifaceted constructs (Morin et al., 2016a). ESEM instead, remains unbiased and produces, in presence of cross-loadings, more exact estimates of factor correlations (Asparouhov et al., 2015). Moreover, bifactor models provide a more flexible alternative than higher-order factor models to adequately reflect the hierarchical nature of multidimensional constructs (Gignac, 2016; Morin et al., 2016a).

Consistent with Schutte et al. (2013) study, Sense of Purpose dimension concerns key aspects of eudaimonic wellbeing including having a purpose in life and self-knowledge (Ryff, 1989; Steger et al., 2006). Purposeful Personal Expressiveness refers to full engagement in activities considered important to the pursuit of personally objectives and it is related to intrinsic motivation (Ryan et al., 2008) and a virtue ethics perspective on eudaimonia (Fowers, 2012). Effortful Engagement reflects the level of effort invested in life’s activities and it could be related to optimal experience and flow constructs (Nakamura and Csikszentmihalyi, 2009).

The factorial structures proposed by Schutte et al. (2013) and Waterman et al. (2010) were compared with an explorative six-factor model in a sample of Spanish adolescents (Salavera and Usán, 2019). Results supported a model with six factors that explained a cumulative variance of 57.84%. The authors also carried out a CFA of the six-factor model in an additional sample.

### The Present Study

**FIGURE 1:**
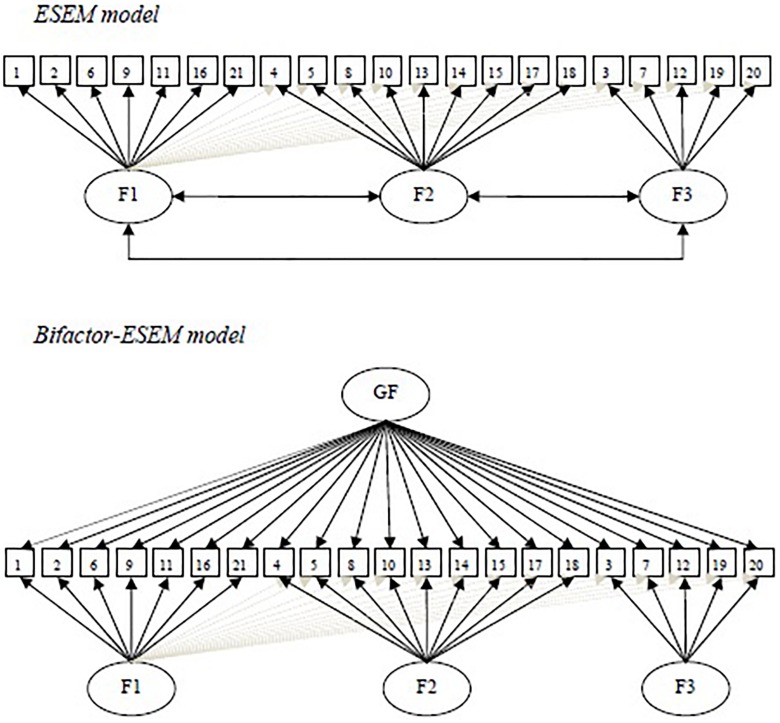
Simplified conceptual representations of estimated models. ESEM cross-loadings (in the ESEM models all items are allowed to cross-load on all of the specific-factors) are not included in this figure to avoid cluttering, but simply illustrated for Factor 1. F1, sense of purpose; F2, purposeful personal expressiveness; F3, effortful engagement; GF, global eudaimonic wellbeing factor.

Aim of this study is to support the multidimensional and hierarchical nature of the QEWB (Waterman et al., 2010) in a Spanish sample through the application of the ESEM framework in line with Fadda et al. (2017). In particular, we carry out an ESEM model composed by three correlated dimensions (Sense of Purpose, Purposeful Personal Expressiveness, Effortful Engagement) and a bifactor-ESEM model, composed by a global eudaimonic wellbeing construct and three specific dimensions of wellbeing (see Figure 1). The ESEM allows to integrate the strengths of exploratory and confirmative factor analysis approach, useful for scale validation; bifactor models produce a more accurate representation of the distinct nature of the various eudaimonic well-being dimensions incorporated in this model.

Consistent with previous studies (Waterman et al., 2010; Fadda et al., 2017), we expect positive correlations with indicators of positive psychological functioning like self-esteem. Finally, to examine factorial invariance across gender and to examine differences in mean levels of eudaimonic wellbeing, we carried out multiple group analyses.

## Materials and Methods

### Participants

A sample of university students from the departments of Psychology (*N* = 497; mean age = 20.5; female = 80.2%), Nursing (*N* = 343; mean age = 20.3; female = 79%) and Education (*N* = 123; mean age = 20.9; female = 91%) of the Pontifical University of Salamanca, Spain, was involved in this study (*N* = 589; 161 males and 428 females; mean age = 19.17; *s.d*. = 1.81). During academic hours, the questionnaires were administrated in 30-min group sessions on students who gave their express authorization; the study was conducted in compliance with APA ethical standards, oral informed consent was requested, confidentiality were guaranteed, and the students could withdraw at any time without justification. The protocol was approved by the ethics committee of the Pontifical University of Salamanca (annexed IV, act 13/02/2019).

### Measures

A Spanish version of the QEWB (Waterman et al., 2010) was developed in accordance with back translation procedures.

(1) The QEWB items were translated into Spanish by two independent experts in the field of psychological evaluation. The Italian version was used as reference for the translation in addition to the English version, since Italy and Spain are more similar in terms of culture, language and history.
(2) The items were back-translated into Italian by other translators with an adequate linguistic knowledge and compared to the original questionnaire.
(3) This preliminary version of the Spanish questionnaire was evaluated using a small pilot sample to determine clarity of the items.
(4) To achieve the final version of questionnaire no relevant change to the items was necessary.

QEWB includes 21 items with 7 negatively worded items. Consistent with the Italian version of the instrument, a 6-point Likert response scale (from 1 = *strongly disagree* to 6 = *completely agree*) was used to increase variability in the responses. The Spanish version is available in the Supplementary Material.

Participants also completed the Rosenberg Self-Esteem Inventory (RSEI; Rosenberg, 1965; Spanish version, Atienza et al., 2000), composed by 10 items (4-point response scale, ranging from 1 = *strongly disagree* to 4 = *completely agree*). Cronbach’s alpha value for RSEI in the present sample was 0.865.

### Analysis

Item distribution analysis showed adequate values for kurtosis (range from −0.10 to 2.60) and univariate skewness (range from −0.015 to −1.59; see Supplementary Material). We used, on standardized data, Mplus 7.3 (Muthén and Muthén, 1998–2014) with robust maximum likelihood (MLR), to estimate ESEM and bifactor-ESEM models. In order to deal with the small number of missing data at the item level (0 to 1.5%, *M* = 0.3%), we used Full Information Maximum Likelihood (FIML). The ESEM solution was specified with oblique target rotation (Asparouhov and Muthén, 2009); for the bifactor-ESEM solution we used a bifactor orthogonal target rotation (Reise, 2012), allowing for the estimation of a G-factor. Contrary to the Italian version of the instrument, no correlated uniqueness was necessary for the Spanish adaptation of the QEWB.

To examine factorial invariance across gender we carried out multiple group analyses testing hypothesis more restrictive at every step (Byrne, 2004). Specifically, we examined configural invariance, followed by weak and strong measurement invariance, and finally latent means invariance. Configural invariance requires the same number of factors as well as the same pattern of relations between items and factors over genders. Weak measurement invariance requires all factor loadings to be constrained to be invariant across groups. To compare group means, strong measurement invariance requires, in addition to factor loadings, the same item intercepts over gender. Indeed, to test latent means, strong measurement invariance needs to be assured. Finally, to examine criterion related validity, the latent correlations between dimensions of the QEWB and self-esteem were estimated.

We used several fit indices to evaluate results: the chi square (χ^2^) test of exact fit, the Tucker-Lewis Index (TLI), the comparative fit index (CFI), the root mean square error of approximation (RMSEA) and the RMSEA 90% confidence interval. Finally, as recommended by Morin et al. (2016b), we reported model-based omega coefficients of composite reliability (McDonald, 1970).

## Results

We tested alternative representations of the QEWB: the 3-factor ESEM and the bifactor-ESEM model including one global factor of eudaimonic wellbeing. Fit indices for the bifactor-ESEM model considerably improved in comparison to the simple ESEM solution. Specifically, lower RMSEA and changes in CFI/TLI ≥ 0.010 (see Table 1).

**TABLE 1:** Fit Indices of model tested.

Models	χ^2^	*df*	CFI	TLI	RMSEA	RMSEA C.I.
ESEM	320.897	150	0.913	0.878	0.044	0.037/0.051
Bifactor-ESEM	235.836	132	0.947	0.916	0.037	0.029/0.044
Criterion	604.072	363	0.942	0.926	0.034	0.029/0.038
Configural invariance for gender	411.330	264	0.927	0.883	0.044	0.035/0.052
Weak measurement invariance	476.832	332	0.928	0.909	0.038	0.030/0.046
Strong measurement invariance	507.078	349	0.921	0.905	0.039	0.032/0.046
Invariance of latent means	662.678	383	0.916	0.847	0.050	0.043/0.056

These results are similar to those found in the Italian study (ESEM: χ^2^ = 273.576, *df* = 149, CFI = 0.919, TLI = 0.886, RMSEA = 0.044, RMSEA CI = [0.035/0.052]; bifactor-ESEM: χ^2^ = 210.064, *df* = 131, CFI = 0.949, TLI = 0.918, RMSEA = 0.037, RMSEA CI = [0.028/0.046]) and support the need to take into account a global overarching construct of eudaimonic wellbeing, over and above the specific components.

### ESEM Solution

As shown in Table 2, the factors were well-defined by the presence of target loadings greater than 0.300, with the sole exception of item 1 (λ = 0.167) for Sense of Purpose, items 4 (λ = 0.277) and 5 (λ = 0.264) for Purposeful Personal Expressiveness and item 7 (λ = 0.278) for Effortful Engagement. It should be noted that these items are the weakest of their respective factors in the Italian study as well (item1: λ = 0.245; item 4: λ = 351; item 5: λ = 0.279; item 7: λ = 0.276).

**TABLE 2:** Standardized parameter estimates from the ESEM solution.

Item	F1 (λ)	F2 (λ)	F3 (λ)	δ
1. I find I get intensely involved in many of the things I do each day.	**0.167**	0.293	0.121	0.807
2. I believe I have discovered who I really am.	**0.749**	0.016	−0.111	0.456
6. I believe I know what my best potentials are and I try to develop them whenever possible.	**0.422**	0.140	0.045	0.746
9. I can say that I have found my purpose in life.	**0.795**	0.072	−0.117	0.356
11. As yet, I’ve not figured out what to do with my life. (R)	**0.601**	−0.152	0.291	0.543
16. I am confused about what my talents really are. (R)	**0.421**	−0.147	0.318	0.711
21. I believe I know what I was meant to do in life.	**0.629**	0.075	−0.189	0.591
4. My life is centered around a set of core beliefs that give meaning to my life.	0.165	**0.277**	−0.270	0.856
5. It is more important that I really enjoy what I do than that other people are impressed by it.	0.143	**0.264**	0.100	0.851
8. I feel best when I’m doing something worth investing a great deal of effort in.	0.008	**0.414**	0.028	0.818
10. If I did not find what I was doing rewarding for me, I do not think I could continue doing it.	0.052	**0.318**	0.065	0.866
13. I believe it is important to know how what I’m doing fits with purposes worth pursuing.	−0.098	**0.575**	−0.025	0.706
14. I usually know what I should do because some actions just feel right to me.	0.093	**0.334**	0.022	0.852
15. When I engage in activities that involve my best potentials, I have this sense of really being alive.	0.064	**0.446**	0.041	0.763
17. I find a lot of the things I do are personally expressive for me.	0.084	**0.319**	0.122	0.830
18. It is important to me that I feel fulfilled by the activities that I engage in.	−0.056	**0.602**	0.178	0.567
3. I think it would be ideal if things came easily to me in my life. (R)	0.067	−0.069	**0.323**	0.893
7. Other people usually know better what would be good for me to do than I know myself. (R)	0.235	−0.072	**0.278**	0.857
12. I can’t understand why some people want to work so hard on the things that they do. (R)	0.018	0.110	**0.516**	0.682
19. If something is really difficult, it probably isn’t worth doing. (R)	−0.019	0.190	**0.655**	0.468
20. I find it hard to get really invested in the things that I do. (R)	0.043	0.088	**0.615**	0.565

*(R) Reversed-scores item; λ, standardized factor loading; δ, standardized item uniqueness; in bold the distribution of items on Fadda et al. (2017) factors (i.e., the target factor loadings); F1, sense of purpose; F2, purposeful personal expressiveness; F3, effortful engagement.*

Multiple cross-loadings were present, but they do not seem to interfere with the interpretation of the factors. Only item1 (*I find I get intensely involved in many of the things I do each day; Siento que me implico intensamente en muchas de las cosas que hago cada día*) loaded more on Purposeful Personal Expressiveness than on Sense of Purpose.

Intercorrelations showed positive relations between Sense of Purpose and Purposeful Personal Expressiveness (*r* = 0.355; *s.e.* = 0.049); Sense of Purpose and Effortful Engagement (*r* = 0.226; *s.e.* = 0.044); and Purposeful Personal Expressiveness and Effortful Engagement (*r* = 0.298; *s.e.* = 0.052).

### Bifactor-ESEM

Fit indices for the bifactor-ESEM solution were adequate (see Table 1); the analysis of standardized factor loading showed a G-factor relatively well defined by the items (| λ| = 0.159 to 0.717, *M* = 0.349, ω = 0.972) with the exception of items 4, 8, and 13 that showed weak factor loadings (≤0.200) on the G-factor (see Table 3).

**TABLE 3:** Standardized parameter estimates from the Bifactor-ESEM solution.

Item	GF (λ)	F1 (λ)	F2 (λ)	F3 (λ)	δ
1. I find I get intensely involved in many of the things I do each day.	**0.268**	**0.186**	0.280	0.159	0.790
2. I believe I have discovered who I really am.	**0.562**	**0.438**	−0.035	−0.180	0.459
6. I believe I know what my best potentials are and I try to develop them whenever possible.	**0.717**	−**0.076**	0.001	−0.218	0.433
9. I can say that I have found my purpose in life.	**0.493**	**0.688**	0.070	−0.085	0.271
11. As yet, I’ve not figured out what to do with my life. (R)	**0.460**	**0.415**	−0.138	0.258	0.530
16. I am confused about what my talents really are. (R)	**0.573**	**0.016**	−0.233	0.121	0.602
21. I believe I know what I was meant to do in life.	**0.356**	**0.510**	0.064	−0.147	0.588
4. My life is centered around a set of core beliefs that give meaning to my life.	**0.175**	0.093	**0.214**	−0.264	0.845
5. It is more important that I really enjoy what I do than that other people are impressed by it.	**0.356**	0.002	**0.201**	0.035	0.831
8. I feel best when I’m doing something worth investing a great deal of effort in.	**0.159**	0.107	**0.388**	0.104	0.801
10. If I did not find what I was doing rewarding for me, I do not think I could continue doing it.	**0.234**	0.024	**0.271**	0.070	0.867
13. I believe it is important to know how what I’m doing fits with purposes worth pursuing.	**0.188**	−0.036	**0.498**	0.032	0.714
14. I usually know what I should do because some actions just feel right to me.	**0.240**	0.072	**0.287**	0.040	0.854
15. When I engage in activities that involve my best potentials, I have this sense of really being alive.	**0.297**	0.034	**0.374**	0.056	0.768
17. I find a lot of the things I do are personally expressive for me.	**0.355**	−0.050	**0.249**	0.059	0.806
18. It is important to me that I feel fulfilled by the activities that I engage in.	**0.328**	−0.028	**0.527**	0.208	0.571
3. I think it would be ideal if things came easily to me in my life. (R)	**0.202**	−0.033	−0.066	**0.243**	0.895
7. Other people usually know better what would be good for me to do than I know myself. (R)	**0.348**	0.018	−0.101	**0.164**	0.841
12. I can’t understand why some people want to work so hard on the things that they do. (R)	**0.300**	−0.030	0.109	**0.465**	0.681
19. If something is really difficult, it probably isn’t worth doing. (R)	**0.354**	−0.032	0.193	**0.616**	0.457
20. I find it hard to get really invested in the things that I do. (R)	**0.368**	−0.034	0.085	**0.537**	0.568

*(R) Reversed-scores item; λ, standardized factor loading; δ, standardized item uniqueness; in bold the distribution of items on Fadda et al. (2017) factors (i.e., the target factor loadings); GF, global factor; F1, sense of purpose; F2, purposeful personal expressiveness; F3, effortful engagement.*

Concerning S-factors, the Purposeful Personal Expressiveness (|λ| = 0.201 to 0.527, *M* = 0.334, ω = 0.944) and the Effortful Engagement (|λ| = 0.164 to 0.616, *M* = 0.405, ω = 0.925) S-factors retained their own specificity in addition to that accounted for by the G-factor, suggesting that they add information to eudaimonic wellbeing G-factor.

In contrast, the Sense of Purpose dimension, and especially two items concerning the perceived development of one’s best potentials (item 6, 16), appeared to retain a weaker specificity if the variance explained by the G-factor was taken into account (|λ| = 0.076 to 0.688, *M* = 0.311, ω = 0.842). Moreover, most of the items of this factor had higher factor loadings on the G-factor (|λ| = 0.268 to 0.717, *M* = 0.490) than the S-factor. This result does not minimize the role of these items; indeed, they mostly serve to reflect students’ global levels of eudaimonic wellbeing, thus supporting the need for a bifactor representation.

### Criterion-Related Validity

To the assessment of the criterion related validity, latent CFA representation of self-esteem was added to the bifactor-ESEM solution (see Table 1).

In line with literature, the global eudaimonic wellbeing (G-factor) was positively correlated with self-esteem measure (*r* = 0.736, *s.d.* = 0.047). With respect to S-factors, Purposeful Personal Expressiveness, Sense of Purpose and Effortful Engagement, no correlation with self-esteem was found.

### Gender Invariance

A lack of decrement in fit indices respect to the recommended cutoff scores (ΔCFI and ΔTLI < 0.01, ΔRMSEA < 0.015), moving from configural to weak and then strong invariance, confirmed the invariance of the QEWS over gender (see Table 1).

Concerning latent means, invariance constraints imposed to the latent means led to a decrement in fit indices, suggesting la presence of not invariant latent means among male and female. The analysis showed that, when males latent means were fixed to zero, females’ latent means were significantly higher on Purposeful Personal Expressiveness (*M* = 0.316, *p* < 0.05) and Effortful Engagement (*M* = 0.663, *p* < 0.01). No significant gender differences appeared on Sense of Purpose and the G-factor latent means.

## Discussion

This study was aimed to support the multidimensional and hierarchical nature of the QEWB in a Spanish sample of university students using a stronger methodological approach. Answers to this Spanish version of QEWB were examined to this purpose using the ESEM framework. Indeed, ESEM integrates the strengths of EFA and CFA approach (Asparouhov and Muthén, 2009); moreover, we also applied bifactor-ESEM, which allows items to load simultaneously on specific (S-factors) components and on a overarching construct (G-factor; Morin et al., 2018).

Results have supported adequate levels of composite reliability and have showed an improved level of fit to the data for the bifactor-ESEM representation of QEWB, in comparison to the ESEM model. In particular, the combination of three specific factors (Sense of Purpose, Purposeful Personal Expressiveness, Effortful Engagement), identified by Schutte et al. (2013) in South African university students, coexisting with a global overarching construct of eudaimonic wellbeing has been confirmed as previously found by Fadda et al. (2017) in an Italian high school students’ sample.

This structure is largely in line with Fadda et al. (2017) study’s results, although some differences appeared, probably due to differences in participants’ age. Specifically, contrary to the Italian version, in the Spanish version, the Purposeful Personal Expressiveness subscale concerning the full engagement in activities subjectively meaningful (e.g., Item 18: *It is important to me that I feel fulfilled by the activities that I engage in; Es importante para mí sentirme realizado en las actividades que llevo a cabo*) appears well-defined, whereas two items forming the Sense of Purpose did not retain a meaningful specificity when the global levels of eudaimonic wellbeing were considered. This is not unusual for bifactor models because the specific dimensions reflect the residual covariance of items after that their shared covariance has been extracted by the G-factor (Morin et al., 2016a). Therefore, our results suggest that Sense of Purpose and in particular items 6 (*I believe I know what my best potentials are and I try to develop them whenever possible; Creo que soy consciente de cuáles son mis mejores cualidades e intento desarrollarlas en la medida de lo posible*) and 16 (*I am confused about what my talents really are; No tengo claro cuáles son mis verdaderos talentos*) concerning the perceived development of one’s best potentials, mostly assess the participants’ global levels of eudaimonic wellbeing. As stated by Waterman et al. (2010) “*It is one thing to have identified one’s talents and skills, but it is another to have decided toward what life goals those talents and skills are to be directed. In order to experience EWB, individuals must find ways for putting their skills and talents to use in the pursuit of personally meaningful objectives* (p. 45).” Probably, in our sample, composed of university rather than high school students, participants have already found the way to pursuit one’s purpose in living and therefore the Sense of Purpose factor lost its specificity, but not the importance of its items in the assessment of the G-factor of eudaimonic wellbeing.

Bifactor-ESEM also allows to assess the criterion-related validity of both the global and specific dimensions (Howard et al., 2018). In the bifactor models, the global and specific factors are orthogonal, therefore, both can freely predict the outcomes, leading a distinct contribute in terms of prediction (Litalien et al., 2017). In line with previous research, our results supported a stronger correlation between the G-factor and self-esteem, whereas specific factors did not presented relationships with this construct once the effect of global eudaimonic wellbeing was considered. Indeed, self-esteem that can be considered the individual’s general evaluation as the global sense of self-worth, is closely related to wellbeing and psycho-social adaptation in adults and adolescents despite differences in culture or nationality (Diener and Diener, 1995; DeNeve and Cooper, 1998).

Finally, tests of gender differences showed that the structure of the QEWB was similar for male and female students. Differences in latent means showed that girls have higher positive Purposeful Personal Expressiveness and Effortful Engagement than boys, whereas no significant differences were found regarding the global eudaimonic wellbeing. Therefore, girls seem to have higher levels on dimensions related to intrinsic motivation and to active engagement in activities considered meaningful (Purposeful Personal Expressiveness), and to work hard into issues, more o less difficult (Effortful Engagement), than boys. However, to fully test generalizability further research is needed to include other age groups and expand the subsample of males. Moreover, a wider array of positive and negative psychological functioning variables are necessary to test convergent and discriminant validity.

## Conclusion

In conclusion, our study supported the idea of a multidimensional representation of the QEWB that reflects probably Waterman et al. (2010)’ criteria to develop the instrument. The authors, in fact, according to eudaimonic wellbeing literature, selected items reflecting six inter-related components of this construct. Moreover, results showed that these items are hierarchically organized around of a global eudaimonic wellbeing component, in line with hierarchical representations of other psychological constructs (e.g., intelligence, self-esteem). This global factor co-exists indeed, with a series of specific dimensions that keep their independence from a more general level of eudaimonic wellbeing.

Therefore, the adoption of this new representation, as captured by the application of the bifactor-ESEM framework, provides a way to reflect a more comprehensive representation of eudaimonic well-being construct.

## Author’s Note

1. We also tested: (1) a one-factor model suggested by Waterman et al. (2010) at the item level in order to avoid parcels, which have been heavily criticized in the absence of a reasonable first-order structure at the item level (Marsh et al., 2013). Results showed inadequate fit indices (χ2 = 1039.449, *df* = 189, CFI = 0.566, TLI = 0.517, RMSEA = 0.087, RMSEA CI = [0.082/0.093]) and factors loadings (range from 0.182 to 0.629, *M* = 0.398); (2) a six-factor solution proposed by Salavera and Usán (2019) based on Waterman et al. (2010) eudaimonic well-being theory. Both, the CFA and the ESEM solutions showed convergence issues.
2. To test whether our linguistic adaptation preserves the psychometric properties found in the Italian validation study of the QEWB, we examined the measurement invariance of the bifactor-ESEM model (which was the best in the Italian study), comparing our sample with 197 university students from Italy (46 males and 151 females; mean age = 21.28; *s.d*. = 1.86). Our results supported invariance of the factor loadings (χ^2^ = 556.026, *df* = 332, CFI = 0.926, TLI = 0.907, RMSEA = 0.041, RMSEA CI = [0.035/0.047]), item thresholds (χ^2^ = 557.971, *df* = 349, CFI = 0.931, TLI = 0.917, RMSEA = 0.039, RMSEA CI = [0.033/0.045]) and latent means (χ^2^ = 607.038, *df* = 383, CFI = 0.926, TLI = 0.919, RMSEA = 0.039, RMSEA CI = [0.033/0.044]) across the Italian and Spanish samples.
3. Although ESEM overcomes restrictions associated with CFA (non-target loadings fixed to zero), on request of one of the paper’s reviewers, we tested a confirmatory solution with three dimensions and a second order factor. As expected, CFA showed inadequate fit indices (χ^2^ = 2167.859, *df* = 210, CFI = 0.806, TLI = 0.780, RMSEA = 0.059, RMSEA CI = [0.053/0.065]) in presence of complex constructs.

## Data Availability Statement

The datasets generated for this study are available on request to the corresponding author.

## Ethics Statement

The studies involving human participants were reviewed and approved by Ethics committee of the Pontifical University of Salamanca. Written informed consent for participation was not required for this study in accordance with the national legislation and the institutional requirements.

## Author Contributions

All authors contributed equally to the theoretical and empirical aspects of the study. MQ-A and MB collected the data. DF analyzed the data and wrote the initial draft of the manuscript. LS supervised the analyses and contributed to the writing of the final version of this manuscript.

## Conflict of Interest

The authors declare that the research was conducted in the absence of any commercial or financial relationships that could be construed as a potential conflict of interest.
