# Supplementary material for: The Multidimensional and Hierarchical Nature of the Questionnaire for Eudaimonic Wellbeing: A Bifactor-ESEM Representation in a Spanish Sample
Source: Front Psychol. 2020 Mar 11;11:422. doi: 10.3389/fpsyg.2020.00422 (PMC7078344; doi:10.3389/fpsyg.2020.00422)
Supplement: Supplementary file 1 [file Table_1.DOCX]

Supplementary Material

# Spanish version of the Questionnaire for Eudaimonic Wellbeing

1. Siento que me implico intensamente en muchas de las cosas que hago cada día.

2. Creo que he descubierto quién soy realmente.

3. Me gustaría que todo fuera simple en mi vida.

4. Mi vida está basada en una serie de creencias que dan sentido a mi existencia.

5. Para mí es más importante disfrutar de lo que hago que impresionar a los demás.

6. Creo que soy consciente de cuáles son mis mejores cualidades e intento desarrollarlas en la medida de lo posible.

7. Otras personas generalmente saben lo que me conviene mejor que yo.

8. Me siento mejor cuando hago algo que requiere mucho esfuerzo.

9. Puedo decir que he encontrado el sentido de mi vida.

10. Si lo que hago no fuera gratificante para mí, creo que no seguiría haciéndolo.

11. Todavía no he descubierto qué debo hacer con mi vida.

12. No entiendo cómo hay personas que ponen tanto empeño en las cosas a las que se dedican.

13. Considero que es importante saber si lo que estoy haciendo me ayuda a alcanzar objetivos que valgan la pena.

14. Generalmente sé cómo debo actuar porque hay cosas que me parece que son claramente adecuadas y correctas.

15. Cuando realizo actividades en las que pongo en práctica mis mejores cualidades, tengo la sensación de estar verdaderamente vivo/viva.

16. No tengo claro cuáles son mis verdaderos talentos.

17. Muchas de las cosas que hago expresan mi personalidad.

18. Es importante para mí sentirme realizado en las actividades que llevo a cabo.

19. Si algo me resulta difícil, probablemente no merezca la pena hacerlo.

20. Me resulta difícil involucrarme en las actividades que realizo.

21. Creo que sé qué es lo que estoy destinado a hacer en la vida.

# Items descriptive statistics

|  | Mean | s.d. | Skewness | Kurtosis |
| --- | --- | --- | --- | --- |
| item1 | 4.53 | 0.897 | -0.665 | 0.671 |
| item2 | 4.10 | 1.081 | -0.484 | 0.265 |
| item3 (R) | 3.84 | 1.450 | -0.389 | -0.733 |
| item4 | 3.15 | 1.371 | -0.015 | -0.936 |
| item5 | 5.16 | 1.044 | -1.591 | 2.605 |
| item6 | 4.51 | 1.008 | -0.763 | 0.746 |
| item7 (R) | 4.11 | 1.301 | -0.422 | -0.575 |
| item8 | 4.68 | 1.025 | -0.642 | 0.193 |
| item9 | 3.89 | 1.172 | -0.369 | -0.100 |
| item10 | 4.92 | 1.252 | -1.201 | 0.825 |
| item11 (R) | 4.16 | 1.382 | -0.394 | -0.759 |
| item12 (R) | 5.15 | 1.099 | -1.430 | 1.715 |
| item13 | 4.93 | 0.919 | -1.097 | 2.079 |
| item14 | 4.70 | 0.896 | -0.633 | 0.696 |
| item15 | 5.09 | 0.842 | -0.850 | 0.680 |
| item16 (R) | 3.70 | 1.280 | -0.039 | -0.550 |
| item17 | 4.93 | 0.857 | -0.848 | 1.134 |
| item18 | 5.11 | 0.853 | -1.046 | 1.586 |
| item19 (R) | 4.97 | 1.157 | -1.365 | 1.727 |
| item20 (R) | 4.48 | 1.224 | -0.584 | -0.401 |
| Item21 | 3.99 | 1.316 | -0.501 | -0.388 |

(R) Reversed-scores item

**
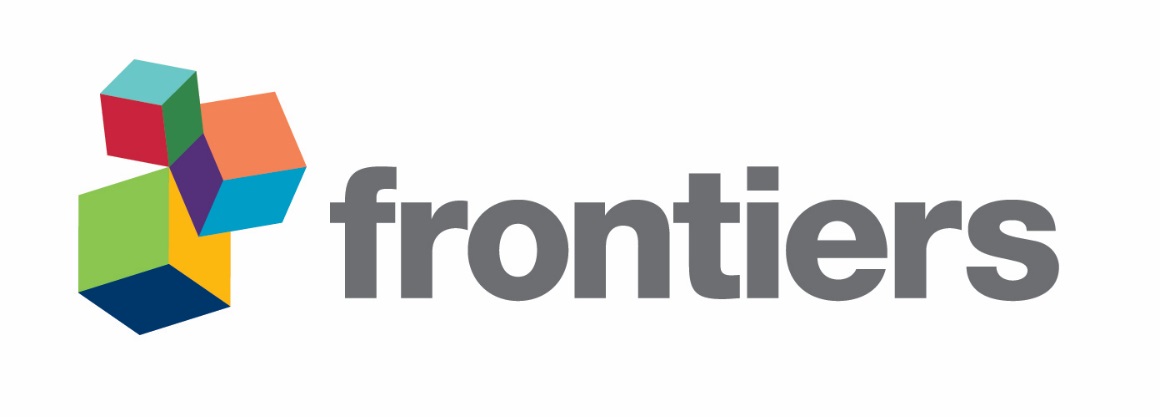
**
